# Multimorbidity and its associated risk factors among older adults in India

**DOI:** 10.1186/s12889-022-13181-1

**Published:** 2022-04-14

**Authors:** Mohd. Rashid Khan, Manzoor Ahmad Malik, Saddaf Naaz Akhtar, Suryakant Yadav, Ratna Patel

**Affiliations:** 1 Department of Health, Government of Mizoram, Aizawl, India; 2 Present Address: Department of Humanities and Social Sciences, Indian Institute of Technology (IIT), Roorkee, Haridwar India; 3 Centre for the Study of Regional Development, School of Social Sciences, Jawaharlal Nehru University, New Delhi, 110067 India; 4 Department of Development Studies, International Institute for Population, Mumbai, India; 5 Department of Public Health and Mortality Studies International Institute for Population Sciences, Mumbai, India

**Keywords:** Multimorbidity, Older adults, Epidemiological transition, Health, Diseases burden

## Abstract

**Background:**

Health at older ages is a key public health challenge especially among the developing countries. Older adults are at greater risk of vulnerability due to their physical and functional health risks. With rapidly rising ageing population and increasing burden of non-communicable diseases older adults in India are at a greater risk for multimorbidities. Therefore, to understand this multimorbidity transition and its determinants we used a sample of older Indian adults to examine multimorbidity and its associated risk factors among the Indian older-adults aged 45 and above.

**Methods:**

Using the sample of 72,250 older adults, this study employed the multiple regression analysis to study the risk factors of multimorbidity. Multimorbidity was computed based on the assumption of older-adults having one or more than one disease risks.

**Results:**

Our results confirm the emerging diseases burden among the older adults in India. One of the significant findings of the study was the contrasting prevalence of multimorbidity among the wealthiest groups (AOR = 1.932; 95% CI = 1.824- 2.032). Similarly women were more likely to have a multimorbidity (AOR = 1.34; 95% CI = 1.282—1.401) as compared to men among the older adults in India.

**Conclusion:**

Our results confirm an immediate need for proper policy measures and health system strengthening to ensure the better health of older adults in India.

**Supplementary Information:**

The online version contains supplementary material available at 10.1186/s12889-022-13181-1.

## Background

India has been witnessing an unprecedented change in the demographic and social structure in recent decades. India is experiencing an epidemiological transition which witnesses a rising burden of noncommunicable diseases (NCDs) [1–3]. NCDs are rapidly increasing in India mainly because of lifestyle changes [1]. With the ageing population in India, which has now become a challenge for public health experts, policymakers, and other research organizations [3–5], the increasing prevalence of senility is a concern in India with the rise of NCDs. There is an urgent need to understand the burden of chronic health conditions among older adults Indians in order to improve and develop suitable responses for the future requirements of healthcare services.

An increase in longevity and decrease in mortality leads to an increase the multiple comorbid conditions, which is commonly known as ‘Multimorbidity.’ In other words, Multimorbidity is defined as the coexistence of two or more chronic conditions which have become prevalent widely [6, 7]. Multimorbidity has now emerged as a major public health issue worldwide, and its associated greater adverse outcome of health like- disability, mortality, poor quality of life, hospitalizations, consequent use of medical resources, and health expenditure [8–11].

Literature suggests that older adults are at larger health risk due to multiple chronic diseases [3, 7, 11–13]. A systematic review study has revealed that the prevalence of multimorbidity among older adults was found to be more than 55% in different countries [14]. Despite that, high multimorbidity prevalence has been observed in many developed nations, for instance- the United States, Australia [13], Canada [15] & Europe [9, 12, 16]. Interestingly, the older adults from developing nations are inadequately equipped with the multimorbidity challenge; as a result, a study conducted in Vietnam [17] revealed that more than 40% of older adults had multimorbidity conditions, whereas 69% in China [11] and 52% in Bangladesh [18]. Besides that, the least developed country like Tanzania, showed 25.3% multimorbidity prevalence among the older population.

### Multimorbidity in Indian settings

Multimorbidity research in India among older adults is still at an early stage. About 23.3% multimorbidity prevalence has been observed in India in the previous study conducted in 2017, where Kerala showed the highest prevalence of multimorbidity with 42%, followed by Punjab (36%), Maharashtra (24%) & West Bengal (23%) [19]. A recent study conducted in the district of Kerala showed 45.4% multimorbidity prevalence [20]. Around 44% multimorbidity prevalence was found in West Bengal [21]. A recent study conducted in the Allahabad district of Uttar Pradesh showed a 31% prevalence of multimorbidity [22].

Literature has suggested that there exists a strong positive association between age and the prevalence of multimorbidity in India [19, 23–25]. A study conducted in Odisha [26] has revealed that multimorbidity prevalence was higher among women than men, and similar results have also been found in West Bengal [21]. The rich older adults in India were more likely to have poor health due to long-term multimorbidity conditions [27]. Recent studies have revealed that there exist significant associations between obesity [28] and loneliness [29] accompanied by multimorbidity in India. Another recent study has investigated in Odisha that multimorbidity increases the odds of older adults' abuse [30]. There are very few studies on multimorbidity prevalence and its associated risk factors among older adults in India. Therefore, we aim to examine the prevalence of multimorbidity and its associated risk factors among older adults in India and its states.

## Methods

### Data source

The data for this study has been taken from Longitudinal Ageing Study in India (LASI) Wave 1, which was carried out during 2017–18. LASI is a multidisciplinary, internationally harmonized panel study of 72,250 older adults aged 45 and above, including their spouses less than 45 years, representative to India and all its states and union territories (excluding Sikkim). It is a baseline data of India’s first longitudinal ageing study that provides a comprehensive scientific evidence base on demographics, household economic status, chronic health conditions, symptom-based health conditions, functional health, mental health (cognition and depression), biomarkers, health insurance, and healthcare utilization, family and social networks, social welfare programs, work and employment, retirement, satisfaction, and life expectations.

### Analytical sample

#### Outcome variables

The outcome variable in the study is multimorbidity which was measured based on multiple chronic diseases reported among the older adults surveyed. Respondents were asked about ten various diseases (See supplementary file), from which the outcome variable of this study was computed. These responses were combined into a trichotomous variable with categories (0 = No), (1 = single), and (2 = more than one morbidity) to study the prevalence. But for regression analysis, we converted the variable into two categories where ‘0’ represented no morbidity, and ‘1’ denoted multimorbidity to apply the logistic model in the study.

#### Independent variables

Demographic and socio-economic risk factors included in the study, such as age, gender, residence, level of education, health insurance status, MPCE (Monthly Per Capita Expenditure) Quintiles, caste-group, religion, currently working, marital status (See supplemental file).

#### Statistical analysis

We used frequencies, percentages, and cross-tabulations for the prevalence of multimorbidity with respect to the social and demographic characteristics with a 95% confidence interval. We applied the chi-square test (χ2) to see the association between multimorbidity and its covariates. We then performed the logistic regression to study the determinants of multimorbidity among older adults in India. All methods were carried out in accordance with relevant guidelines and regulations. Furthermore, this study is based on secondary source of data and therefore authors did not require any informed consent from the participant. However, the survey agencies that collected data obtained prior consent from the participants.

## Results

### Socio-demographic characteristics

Table 1 provides the sample distribution of the older-adults in India. Around 42% older-adults are males as compared to 58 percent females. About 68% of older-adults belong to rural areas as compared to 32% of older-adults in urban area. The sample aged more than 75 years and above is around 8% as compared to 13% aged 45 and below. Poorer groups showed the highest percentage with 21.22% followed by Poorest (20.7%) and Middle (20.47%) while Richest showed lowest with 18.03%. Illiterate older adults have showed highest percentage with 49.5% followed by Primary education level (23.21%) while the lowest educational level is observed among above secondary (10.38%). However, Hindu religion is showing highest percentage with 81.92% followed by Muslims (11.67%), while others religion group showed lowest with only 6.41%. OBC constitutes highest percentage of older adults with 46.72% while Schedule Tribe is found be lowest with only 8.76%. On the other hand, currently working older adults are showing 46.28% while those who never worked comprises of 27.57% and those who are not currently working is showing the lowest percentage with 26.14%. The highest percentage is observed among married older adults with 75.6%, while widowed showed 21.66% and others is found to be lowest with only 2.73%.

**Table 1:** Sample distribution of older adults in India by various socio-demographic and economic background (*N* = 72,250)

Background	Numbers	Percentage (%)
***Place of Residence***		
Rural	49,274	68.2
Urban	22,976	31.8
***MPCE quintile***		
Poorest	14,956	20.7
Poorer	15,328	21.22
Middle	14,790	20.47
Richer	14,151	19.59
Richest	13,025	18.03
***Age at last birthday***		
≥ 45 Years	9168	12.69
46–60 Years	33,115	45.83
61–75 Years	24,002	33.22
Above 75 Years	5965	8.26
***Highest level of Education***		
Illiterate	35,763	49.5
Primary	16,771	23.21
Secondary/Matriculation	12,216	16.91
Above Secondary	7499	10.38
***Religion***		
Hindu	59,186	81.92
Muslim	8428	11.67
Others	4631	6.41
***Caste Category***		
Scheduled caste	13,688	19.66
Scheduled tribe	6102	8.76
Other backward class	32,527	46.72
None of them	17,310	24.86
***Sex of Respondent***		
Male	30,342	42
Female	41,908	58
***Currently working***		
Yes	33,431	46.28
No	18,884	26.14
Never worked	19,915	27.57
***Current Marital Status***		
Currently married	54,621	75.6
Widowed	15,650	21.66
Others	1975	2.73
**Total**	**72,250**	**100**

Source: Authors’ calculation using LASI-wave-1 data

### Multimorbidity prevalence at the national level

Table 2 shows that the prevalence of multimorbidity increased substantially with age, from 48.31% (95% CI = 46.01%—50.61%) in ≥ 45 years to 73.86% (95% CI = 71.44%—76.14%) in those aged above 75 years. Similarly, the crude prevalence of multimorbidity increased modestly with increasing household wealth, from 53.78% (95% CI = 52.32%—55.23%) in the lowest wealth quintile to 71.97% (95% CI = 70.02%—73.84%) in the highest wealth quintile. There is considerably higher prevalence of multimorbidity in urban areas [69.59% (95% CI = 67.66%—71.45%)] as against in rural area [59.48% (95% CI = 58.79%—60.17%)]. There seems not much difference in male and female as far as multimorbidity is concern, however, widowed has higher prevalence of multimorbidity 69.51% (95% CI = 67.98%—71.00%) as compared to currently married 60.92% (95% CI = 60.04%—61.78%).

**Table 2:** Prevalence of morbidity (%) among the older adults in India with suitable socio-demographic and economic background (*N* = 72,250)

Background	No-Morbidity	Single-Morbidity	Multimorbidity
	**[95% C.I.]**	**[95% C.I.]**	**[95% C.I.]**
***Place of Residence***			
Rural	20.65 (20.07–21.23)	19.88 (19.32–20.44)	59.48 (58.79–60.17)
Urban	14.08 (12.7–15.58)	16.33 (14.94–17.84)	69.59 (67.66–71.45)
***MPCE quintile***			
Poorest	24.86 (23.57–26.2)	21.36 (20.15–22.63)	53.78 (52.32–55.23)
Poorer	19.79 (18.75–20.88)	19.33 (18.29–20.42)	60.87 (59.53–62.19)
Middle	18.13 (17.03–19.29)	19.68 (17.98–21.5)	62.19 (60.42–63.92)
Richer	16.34 (14.57–18.27)	17.68 (16.57–18.84)	65.99 (64.08–67.85)
Richest	12.82 (11.66–14.08)	15.21 (13.98–16.54)	71.97 (70.02–73.84)
***Age at last birthday***			
≥ 45 Years	30.75 (28.86–32.70)	20.95 (19.61–22.36)	48.31 (46.01–50.61)
46–60 Years	21.10 (20.09–22.15)	19.67 (18.65–20.73)	59.23 (57.93–60.51)
61–75 Years	12.71 (11.93–13.54)	17.11 (16.29–17.96)	70.18 (69.06–71.27)
Above 75 Years	9.24 (7.85–10.86)	16.90 (14.95–19.04)	73.86 (71.44–76.14)
***Highest level of Education***			
Illiterate	20.95 (20.21–21.71)	19.71 (18.98–20.46)	59.34 (58.40–60.28)
Primary	16.37 (15.39–17.39)	17.47 (16.58–18.00)	66.17 (64.93–67.38)
Secondary/Matriculation	15.52 (14.44–16.67)	18.19 (16.88–19.58)	66.29 (64.50–68.03)
Above Secondary	17.04 (13.70–20.99)	17.98 (14.76–21.72)	64.99 (60.39–69.32)
***Religion***			
Hindu	19.13 (18.53–19.73)	19.12 (18.46- 19.80)	61.75 (60.92–62.58)
Muslim	15.59 (14.02–17.00)	16.88 (15.15–18.77)	67.53 (65.10–69.87)
Others	16.77 (12.44–22.23)	17.42 (15.39–19.64)	65.81 (61.47–69.91)
***Caste Category***			
Scheduled caste	19.46 (18.39–20.58)	20.24 (19.16–21.37)	60.30 (58.92–61.66)
Scheduled tribe	29.78 (28.14–31.47)	20.65 (19.19–22.18)	49.58 (47.66–51.49)
Other backward class	18.48 (17.37–19.63)	18.84 (17.76–19.98)	62.68 (61.21–64.13)
None of them	14.39 (13.60–15.22)	17.01 (16.16–17.89)	68.6 (67.53–69.66)
***Sex of Respondent***			
Male	18.3 (17.53–19.09)	19.4 (18.58–20.25)	62.30 (61.20–63.38)
Female	18.76 (17.89–19.66)	18.29 (17.46–19.15)	62.95 (61.85–64.03)
***Currently working***			
Yes	23.42 (22.59–24.27)	20.63 (19.83–21.45)	55.96 (54.83–57.07)
No	11.12 (10.28–12.02)	16.74 (15.79–17.73)	72.15 (70.91–73.35)
Never worked	17.45 (15.99–19.02)	17.52 (16.11–19.01)	65.03 (63.20–66.82)
***Current Marital Status***			
Currently married	19.75 (19.14–20.38)	19.33 (18.62–20.06)	60.92 (60.04–61.78)
Widowed	13.53 (12.45–14.69)	16.96 (15.89–18.08)	69.51 (67.98–71.00)
Others	25.83 (17.03–37.15)	17.09 (14.08–20.59)	57.08 (48.73–65.04)
**Total**	**18.57 (17.96–19.19)**	**18.75 (18.16–19.36)**	**62.68 (61.90–63.45)**

Source: Authors’ calculation using LASI-wave-1 data; Abbreviation: *CI *Confidence interval

### Single & multimorbidity prevalence at the state level

**Fig. 1:**
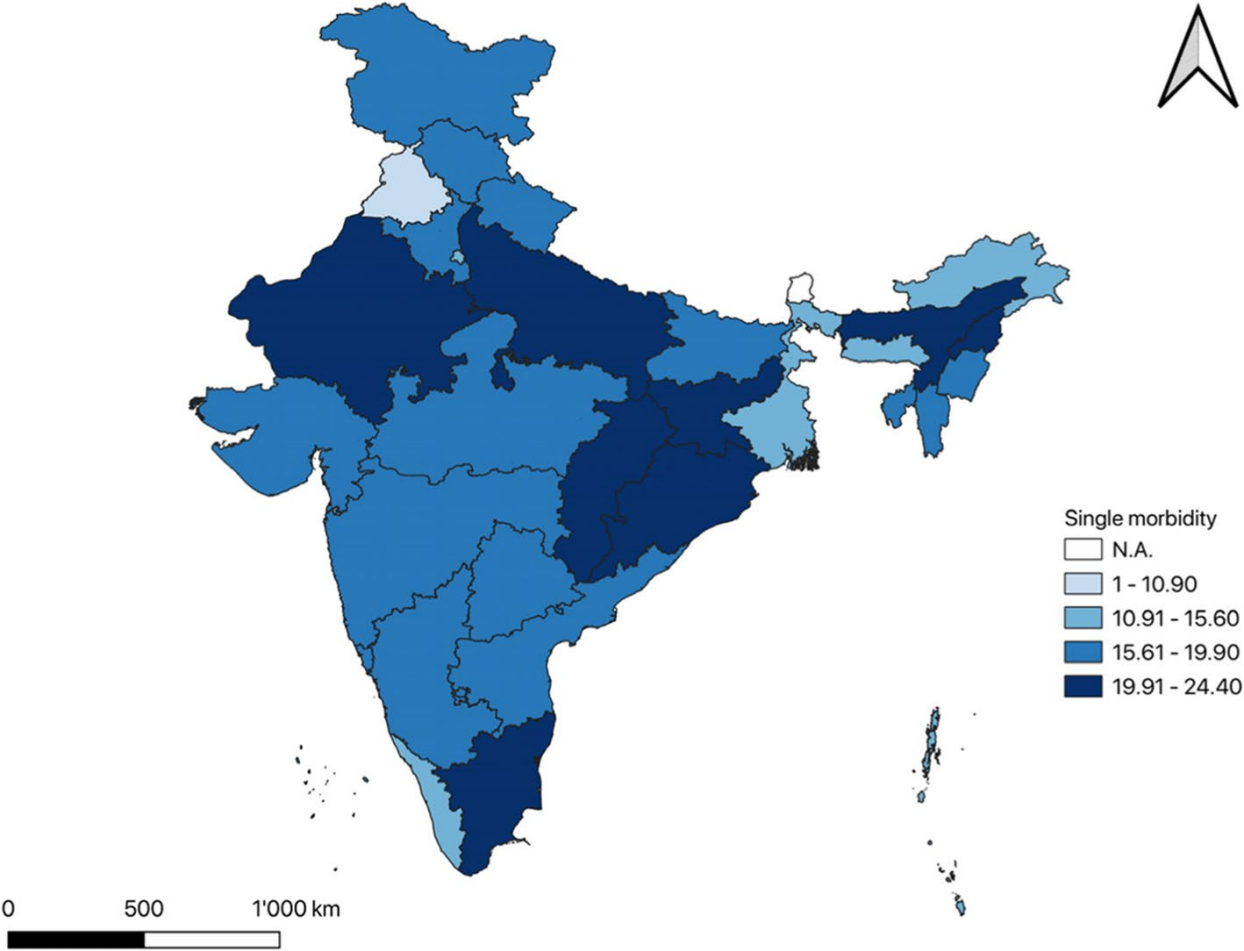
Single morbidity prevalence among the older adults in India at state level using LASI Wave-1 data

**Fig. 2:**
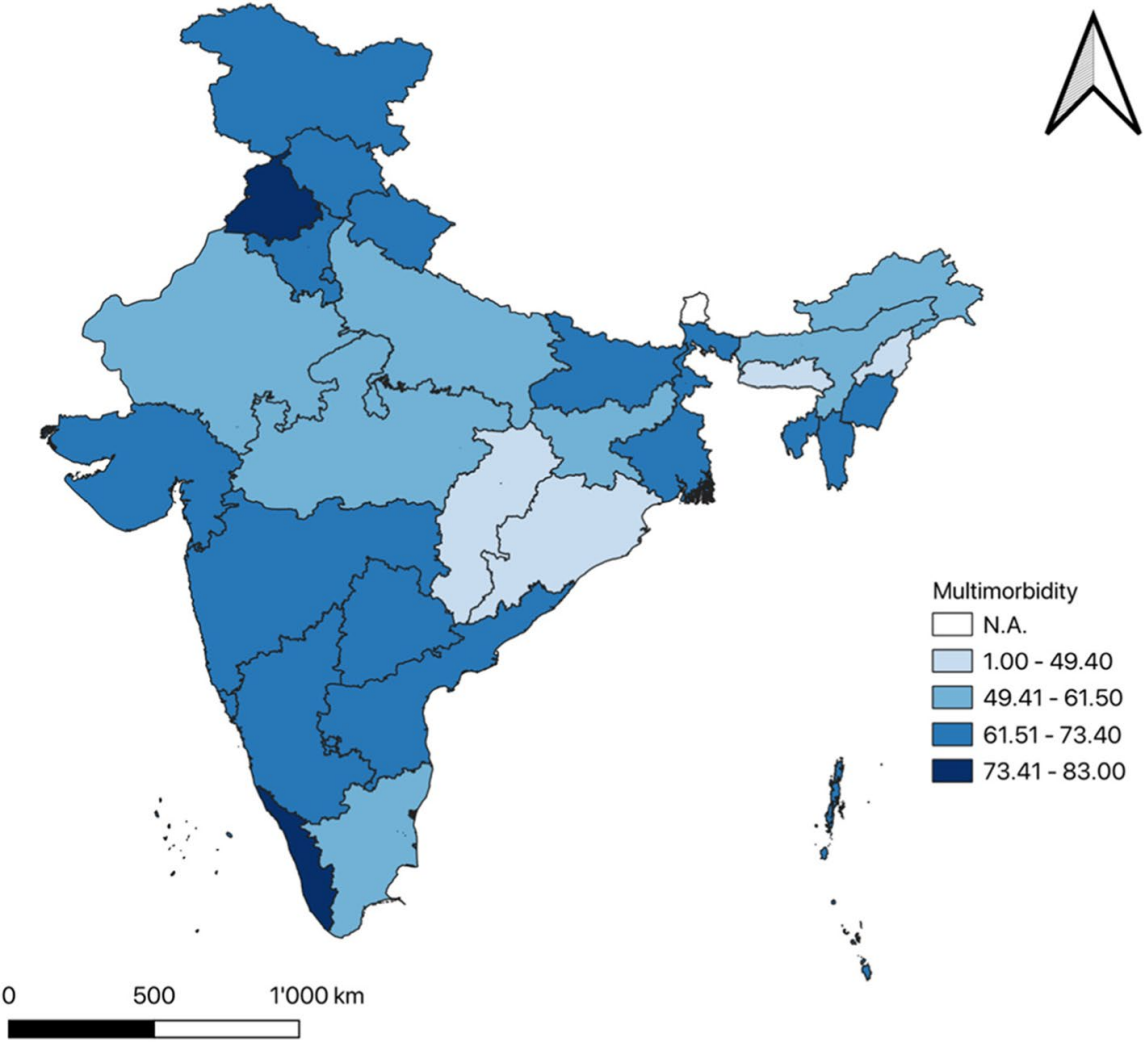
Multimorbidity prevalence among the older adults in India at state level using LASI Wave-1 data

Figure 1 shows the single morbidity prevalence among the older adults in India at the state level using LASI Wave-1 data. The highest single morbidity prevalence was in Odisha (24.4%), followed by Assam (23.2%), Chhattisgarh (23%) & Tamil Nadu (22%), whereas the lowest was seen in Punjab (10.9%), and followed by Kerala (13.4%), Meghalaya (14%) & Chandigarh (14%). Figure 2 indicates the multimorbidity prevalence among older adults in India at the state level. The prevalence of multimorbidity was the highest in Punjab (83%), Chandigarh (78.7%), Kerala (78%), West Bengal (73.4%), & Goa (72.5%), while the lowest was found in Nagaland with 42.6%, followed by Chhattisgarh (44.6%), Meghalaya (48.8%), Odisha (49.4%) & Jharkhand (51.5%).

### Determinants of multimorbidity

Table 3 depicts the result of regression analysis. The predicted probability of having multiple diseases showed a significant increase, and it is almost three times more likely in 75 years and above (Adjusted OR = 3.325; 95% CI = 3.05 – 3.624; reference category ≥ 45 years of age). Women were more likely than men to have more than one morbidity (Adjusted OR = 1.34; 95% CI = 1.282—1.401). Other characteristics like urban resident (OR = 1.408; 95% CI = 1.355—1.460) with reference to rural, Muslim (AOR = 1.307; 95% CI = 1.237—1.382) against Hindu, and widowed (AOR = 1.08; 95% CI = 1.031—1.131) against currently married have higher likelihood of having multimorbidity. Risk of multimorbidity was highest among the better affluent groups (Richest) (AOR = 1.932; 95% CI = 1.824- 2.032) as compared to the older adults belonging to poorer households who are having the lower odds of having any multimorbidity (AOR = 1.244; 95% CI = 1.183—1.307).

**Table 3:** Regression analysis result showing the associated risk factors of multimorbidity among the older adults in India (*N* = 72,250)

Background	Adjusted Odds Ratio	Confidence Interval [95%]
***Place of Residence***		
Rural^c^		
Urban	1.408^a^	[1.355,1.46]
***MPCE quintile***		
Poorest^c^		
Poorer	1.244^a^	[1.183,1.307]
Middle	1.375^a^	[1.313,1.452]
Richer	1.629^a^	[1.55,1.719]
Richest	1.932^a^	[1.824,2.032]
***Age at last birthday***		
≥ 45 Years^c^		
46–60 Years	1.971^a^	[1.875,2.072]
61–75 Years	3^a^	[2.831,3.178]
Above 75 Years	3.325^a^	[3.05,3.624]
***Highest level of Education***		
Illiterate^c^		
Primary	1.34^a^	[1.285,1.398]
Secondary/Matriculation	1.321^a^	[1.258,1.387]
Above Secondary	1.202^a^	[1.127,1.283]
***Religion***		
Hindu^c^		
Muslim	1.307^a^	[1.237,1.382]
Others	1.207^a^	[1.146,1.272]
***Caste Category***		
Scheduled caste^c^		
Scheduled tribe	0.884^a^	[0.839,0.932]
Other backward class	0.564^a^	[0.533,0.596]
None of them	0.857^a^	[0.821,0.894]
***Sex of Respondent***		
Male^c^		
Female	1.34^a^	[1.282,1.401]
***Currently working***		
Yes^c^		
No	1.489^a^	[1.423,1.558]
Never worked	1.124^a^	[1.075,1.175]
***Current Marital Status***		
Currently married^c^		
Widowed	1.08^a^	[1.03,1.131]
Others	0.946	[0.863,1.037]
***Alcohol consumption (ever)***		
No^c^		
Yes	1.195^a^	[1.14,1.254]

Source: Authors’ calculation using LASI-wave-1 data

95% confidence interval in the parentheses. Significant level at: ^a^significant at 1 percent and ^b^significant at 5 percent, ® is the reference category of the independent variables

## Discussion

Multimorbidity is emerging as a critical public health challenge, especially in developing countries such as India. Owing to the lifestyle changes, shift in disease patterns, and rise in out-of-pocket expenditure (OOPE), multimorbidity is resulting in an economic burden for countries. In parallel to the rise in multimorbidity, the ageing of the population with an increase in life expectancy has become a major public health challenge. The ageing of the population further manifests the multifold vulnerability in old ages caused by these diseases’ risks. In view of the rise in the risk of diseases, we examined the prevalence and risk factors of multimorbidity in 45 and above years using data provided in the LASI wave-1 in India. Our results clearly showed the greater risk for multimorbidity among the older adults being vulnerable in terms of socio-economic hierarchy. Elderly belonging to lower socio-economic groups are at higher risk for multimorbidity, however we found contrasting results where elderly from better socio-economic groups were at a greater risk for multimorbidities in India.

An individual suffers from multimorbidity due to multiple reasons ranging from comorbidities that may arise due to a common risk factor or due to the outcome of a particular disease leading to other diseases [31]. This risk likely enhances with age due to physical and functional vulnerabilities. Research shows ageing contributes to multimorbidity through the loss of physical and functional health, including frailty, which later results in greater complications like falls, disability, immobility, and mortality [32, 33].

Our results showed the significant association between multimorbidity and its associated demographic and socioeconomic risk factors like age, income, education, and place of residence. The results corroborate with the earlier findings where a significant association was found between multimorbidity and socio-economic outcomes [34].

This study showed the significant and positive relation of multimorbidity in urban areas. The risk associated with multimorbidity is higher in 45 and above years in urban areas as compared to rural areas. This higher risk in urban areas is likely attributable to increasing lifestyle changes [35]. This higher risk of disease in urban areas is also appreciated due to the imbalance in medical care that exists in weak health care facilities [36].

One of the significant findings of this paper is the contrasting prevalence of multimorbidity among the wealthiest groups, which diverges from some earlier studies from developing countries examining multimorbidity [37, 38]. One of the most likely reasons may be self-reporting of morbidity, given the fact that older adults belonging to better socio-economic classes have greater access to health care service provisions, which increases the likelihood of their diagnosis and care for a particular disease [39].

Multimorbidity increases likely ageing risk, as shown by various studies [33, 40]. These findings are also well reflected through our results, where an increase in age likely enhances the risk for more than one morbidity. Therefore, increasing longevity has likely consequences of morbidity patterns of older adults, which needs immediate policy attention to avert the challenges of morbidity, disability, and death at older ages. Furthermore, strong measures can ensure active and healthy ageing interventions to avert the burden of the disease with a greater concentration of older adults in upper ages.

## Conclusions

This study provides evidence of emerging diseases burden among older adults in India. The study highlights the need for better interventions for older adults to avert the health crisis in later years of life. As the findings of this research specifically indicate the growing burden of multimorbidity, there is an immediate need for proper policy measures and health system strengthening to ensure health ageing in India. Moreover, emphasis should be given to workforce training and quality improvement strategies that can ensure the better physical and functional health of older adults. There is also an immediate need for improving the financial incentives for older adults at older ages, given the challenges they face in terms of health and social security provisions in India.

### Limitations of the study

Our study has several limitations. Our study is based on cross-sectional data therefore we could not able to establish causality. We have not included 'Sikkim' state in the study because of data unavailability. The existing data only contains information on prevalence and determinants, which limits our understanding of the severity of diseases and multimorbidity. Furthermore, our study did not include lifestyle factor, dietary and personal habits, as these information were not available in the survey data.

## Supplementary Information

**Additional file 1.**

## Data Availability

The datasets generated and/or analysed during the current study are available with the International Institute for Population Sciences, Mumbai, India repository and could be accessed from the following link: https://iipsindia.ac.in/sites/default/files/LASI_DataRequestForm_0.pdf. Those who wish to download the data have to follow the above link. This link leads to a data request form designed by International Institute for Population Sciences. After completing the form, it should be mailed to: datacenter@iips.net for further processing. After successfully sending the mail, individual will receive the data in a reasonable time.

## Abbreviations

LASI: Longitudinal Ageing Study in India
MPCE: Monthly Per Capita Expenditure
NCD: Non-Communicable Diseases
OOPE: Out-of-Pocket Expenditure
NA: Not available

## References

[1] Quigley MA (2006). Commentary: Shifting burden of disease—epidemiological transition in India. Int J Epidemiol.

[2] Yadav S, Arokiasamy P (2014). Understanding epidemiological transition in India. Glob Health Action.

[3] Arokiasamy P, Uttamacharya U, Jain K, Biritwum RB, Yawson AE, Wu F (2015). The impact of multimorbidity on adult physical and mental health in low- and middle-income countries: what does the study on global ageing and adult health (SAGE) reveal?. BMC Med.

[4] Abegunde DO, Mathers CD, Adam T, Ortegon M, Strong K (2007). The burden and costs of chronic diseases in low-income and middle-income countries. The Lancet.

[5] Akhtar SN, Saikia N. Differentials and predictors of hospitalisation among the elderly people in India: evidence from 75th round of National Sample Survey (2017-2018). Working with Older People. 2022.

[6] Johnston MC, Crilly M, Black C, Prescott GJ, Mercer SW (2019). Defining and measuring multimorbidity: a systematic review of systematic reviews. Eur J Public Health..

[7] Salive ME (2013). Multimorbidity in older adults. Epidemiol Rev.

[8] Bähler C, Huber CA, Brüngger B, Reich O (2015). Multimorbidity, health care utilization and costs in an elderly community-dwelling population: a claims data based observational study. BMC Health Serv Res.

[9] Garin N, Olaya B, Moneta MV, Miret M, Lobo A, Ayuso-Mateos JL (2014). Impact of multimorbidity on disability and quality of life in the Spanish older population. PLoS ONE..

[10] McPhail S (2016). Multimorbidity in chronic disease: impact on health care resources and costs. RMHP.

[11] Zhang L, Ma L, Sun F, Tang Z, Chan P (2020). A Multicenter Study of Multimorbidity in Older Adult Inpatients in China. J Nutr Health Aging.

[12] Bezerra de Souza DL, Oliveras-Fabregas A, Espelt A, Bosque-Prous M, de Camargo Cancela M, Teixidó-Compañó E (2021). Multimorbidity and its associated factors among adults aged 50 and over: a cross-sectional study in 17 European countries. PLoS ONE..

[13] Walker AE (2007). Multiple chronic diseases and quality of life: patterns emerging from a large national sample. Aust Chronic Illn.

[14] Marengoni A, Angleman S, Melis R, Mangialasche F, Karp A, Garmen A (2011). Aging with multimorbidity: A systematic review of the literature. Ageing Res Rev.

[15] Geda NR, Janzen B, Pahwa P (2021). Chronic disease multimorbidity among the Canadian population: prevalence and associated lifestyle factors. Arch Public Health.

[16] Willadsen TG, Bebe A, Køster-Rasmussen R, Jarbøl DE, Guassora AD, Waldorff FB (2016). The role of diseases, risk factors and symptoms in the definition of multimorbidity – a systematic review. Scand J Prim Health Care.

[17] Ha NT, Le NH, Khanal V, Moorin R (2015). Multimorbidity and its social determinants among older people in southern provinces. Vietnam Int J Equity Health.

[18] Khanam MA, Streatfield PK, Kabir ZN, Qiu C, Cornelius C, Wahlin Å (2011). Prevalence and Patterns of Multimorbidity among Elderly People in Rural Bangladesh: A Cross-sectional Study. J Health Popul Nutr.

[19] Talukdar B, Himanshu H. Prevalence of multimorbidity (chronic NCDS) and associated determinants among elderly in India. Demography India. 2017:69–76.

[20] Rohini C, Jeemon P (2020). Prevalence and patterns of multi-morbidity in the productive age group of 30–69 years: a cross-sectional study in Pathanamthitta District. Kerala. Wellcome Open Res.

[21] Vadrevu L, Kumar V, Kanjilal B (2016). Rising challenge of multiple morbidities among the rural poor in India-a case of the Sundarbans in West Bengal. Int J Med Sci Public Health.

[22] Verma V, Mishra N (2019). A study on multi-morbidity among geriatric group in a District of Northern India: a cross sectional study. IJMEDPH.

[23] Banjare P, Pradhan J (2014). Socio-economic inequalities in the prevalence of multi-morbidity among the rural elderly in Bargarh District of Odisha (India). PLoS ONE..

[24] Britt HC, Harrison CM, Miller GC, Knox SA (2008). Prevalence and patterns of multimorbidity in Australia. Med J Aust.

[25] Guralnik JM (1996). Assessing the impact of comorbidity in the older population. Ann Epidemiol.

[26] Kshatri JS, Palo SK, Bhoi T, Barik SR, Pati S (2020). Prevalence and patterns of multimorbidity among rural elderly: findings of the AHSETS study. Front Public Health..

[27] Irshad CV, Dash U. Healthy aging in India: evidence from a panel study. J Health Res. 2021.

[28] Srivastava S, Joseph KJV, Dristhi D, Muhammad T (2021). Interaction of physical activity on the association of obesity-related measures with multimorbidity among older adults: a population-based cross-sectional study in India. BMJ Open..

[29] Ansari S, Muhammad T, Dhar M (2021). How does multi-morbidity relate to feeling of loneliness among older adults? evidence from a population-based survey in India. Popul Ageing.

[30] Kshatri JS, Bhoi T, Barik SR, Palo SK, Pati S (2021). Is multimorbidity associated with risk of elder abuse? Findings from the AHSETS study. BMC Geriatr.

[31] Neale MC, Kendler KS (1995). Models of comorbidity for multifactorial disorders. Am J Hum Genet.

[32] Fried LP, Ferrucci L, Darer J, Williamson JD, Anderson G (2004). Untangling the concepts of disability, frailty, and comorbidity: implications for improved targeting and care. J Gerontol A Biol Sci Med Sci.

[33] Barnett K, Mercer SW, Norbury M, Watt G, Wyke S, Guthrie B (2012). Epidemiology of multimorbidity and implications for health care, research, and medical education: a cross-sectional study. The Lancet..

[34] Pathirana TI, Jackson CA (2018). Socioeconomic status and multimorbidity: a systematic review and meta-analysis. Aust N Z J Public Health.

[35] Li Q, Hsia J, Yang G (2011). Prevalence of Smoking in China in 2010. N Engl J Med.

[36] Wang R, Yan Z, Liang Y, Tan ECK, Cai C, Jiang H (2015). Prevalence and patterns of chronic disease pairs and multimorbidity among older chinese adults living in a rural area. PLoS ONE..

[37] Fried LP, Ferrucci L, Darer J, Williamson JD, Anderson G. Untangling the concepts of disability, frailty, and comorbidity: implications for improved targeting and care. The Journals of Gerontology Series A: Biological Sciences and Medical Sciences. 2004;59(3):M255–63. 10.1093/gerona/59.3.m255 15031310

[38] Hosseinpoor AR, Bergen N, Mendis S, Harper S, Verdes E, Kunst A (2012). Socioeconomic inequality in the prevalence of noncommunicable diseases in low- and middle-income countries: results from the world health survey. BMC Public Health..

[39] Vellakkal S, Subramanian SV, Millett C, Basu S, Stuckler D, Ebrahim S (2013). Socioeconomic inequalities in non-communicable diseases prevalence in india: disparities between self-reported diagnoses and standardized measures. PLoS ONE..

[40] Wolff JL, Starfield B, Anderson G (2002). Prevalence, expenditures, and complications of multiple chronic conditions in the elderly. Arch Intern Med.

